# Supplementary material for: Inflammatory factors and risk of lung adenocarcinoma: a Mendelian randomization study mediated by blood metabolites
Source: Front Endocrinol (Lausanne). 2024 Aug 27;15:1446863. doi: 10.3389/fendo.2024.1446863 (PMC11384989; doi:10.3389/fendo.2024.1446863)
Supplement: Supplementary file 1 [file DataSheet1.zip › supplementary files/S2/GCST90200409/forest.pdf]

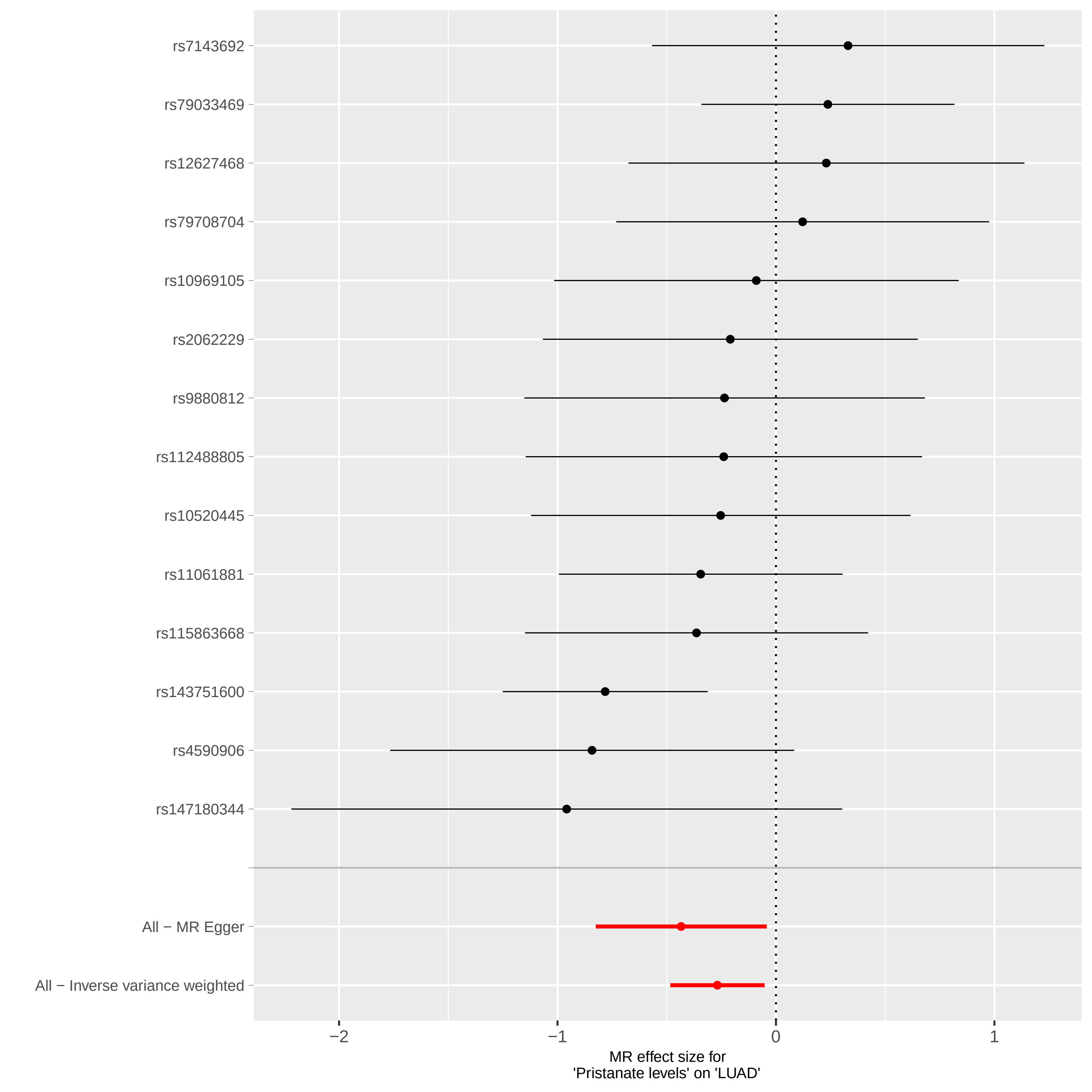

rs7143692
rs79033469
rs12627468
rs79708704
rs10969105
rs2062229
rs9880812
rs112488805
rs10520445
rs11061881
rs115863668
rs143751600
rs4590906
rs147180344
All − MR Egger
All − Inverse variance weighted
−2
−1
0
1
MR effect size for
'Pristanate levels' on 'LUAD'
